# Postoperative Acute Kidney Injury in Cardiovascular Surgery Patients with Prolonged Intensive Care Unit Stay: Impact on Mortality and Outcomes

**DOI:** 10.21470/1678-9741-2025-0271

**Published:** 2026-09-10

**Authors:** Bedih Balkan, Engin Ihsan Turan, Ahmet Said Dündar, Lokman Yalçın

**Affiliations:** 1 Department of Anesthesiology and Reanimation, Intensive Care, Kanuni Sultan Süleyman Training and Research Hospital, Health Sciences University, Istanbul, Turkiye; 2 Department of Anesthesiology and Reanimation, Kanuni Sultan Süleyman Training and Research Hospital, Health Sciences University, Istanbul, Turkiye; 3 Department of Internal Medicine, Kanuni Sultan Süleyman Training and Research Hospital, Health Sciences University, İstanbul, Turkiye; 4 Department of Cardiovascular Surgery, Mehmet Akif Ersoy Thoracic and Cardiovascular Surgery Training and Research Hospital, Istanbul, Türkiye

**Keywords:** Kidney (Renal Function, Failure, Dialysis), Cardiac (Use in Combination), Postoperative Care.

## Abstract

**Objective:**

To evaluate the association between postoperative acute kidney injury (AKI) and outcomes in adults undergoing cardiovascular surgery who required a prolonged intensive care unit (ICU) stay.

**Methods:**

We conducted a retrospective cohort study of consecutive cardiovascular surgery patients with ICU stay ≥ 72 hours. AKI was defined and staged using creatinine-based Kidney Disease: Improving Global Outcomes (KDIGO) criteria. The primary outcome was in-hospital mortality. Secondary outcomes were time to extubation, ICU days until ward transfer, and perioperative transfusion exposure (red blood cells/fresh frozen plasma/platelet/apheresis); initiation of renal replacement therapy was assessed exploratorily. Multivariable logistic regression examined the association between AKI and mortality, adjusting for age, sex, baseline renal function/chronic kidney disease (estimated glomerular filtration rate category), hypertension, diabetes, chronic obstructive pulmonary disease, ejection fraction (or Age Creatinine Ejection Fraction score), procedure type (coronary artery bypass grafting/valve/combined), cardiopulmonary bypass and cross-clamping times, transfusions, and post-cardiopulmonary bypass vasoactive/inotropic use where available.

**Results:**

AKI was frequent after cardiovascular surgery and was independently associated with higher in-hospital mortality in adjusted analyses. Patients with AKI also had longer time to extubation, greater transfusion requirements, and prolonged ICU stay, with a stepwise worsening across KDIGO stages. Findings were consistent in sensitivity analyses. Incomplete urine-output data were acknowledged as a limitation, as oliguria forms part of KDIGO definitions.

**Conclusion:**

Among cardiovascular surgery patients with prolonged ICU stay, postoperative AKI is a strong predictor of in-hospital mortality and adverse resource-related outcomes. Emphasis on prevention, early detection, and optimized perioperative management may improve prognosis in this high-risk population.

## INTRODUCTION

**Table:** 

Abbreviations, Acronyms & Symbols				
ACC	= Aortic cross-clamping		ES	= Erythrocyte suspension
ACEF	= Age, Creatinine, Ejection Fraction		FFP	= Fresh frozen plasma
AKI	= Acute kidney injury		HT	= Hypertension
AKIN	= Acute Kidney Injury Network		ICU	= Intensive care unit
ANOVA	= Analysis of variance		IRB	= Institutional Review Board
B	= Regression coefficient		KDIGO	= Kidney Disease: Improving Global Outcomes
BMI	= Body mass index		NSAIDs	= Nonsteroidal anti-inflammatory drugs
CABG	= Coronary artery bypass grafting		OR	= Odds ratio
COPD	= Chronic obstructive pulmonary disease		RBC	= Red blood cells
CPB	= Cardiopulmonary bypass		RIFLE	= Risk, Injury, Failure, Loss, and End-stage kidney disease
df	= Degrees of freedom		RRT	= Renal replacement therapy
DM	= Diabetes mellitus		S.E.	= Standart error
EF	= Ejection fraction			

Coronary artery disease and valvular heart disease remain the leading causes of morbidity and mortality worldwide. Cardiovascular surgeries, including coronary artery bypass grafting (CABG) and valve replacement surgeries, have become standard treatments for patients with advanced stages of these conditions^[^1^]^. These procedures support cardiovascular function by improving myocardial perfusion and correcting valvular abnormalities, thereby enhancing patients' quality of life. However, cardiovascular surgeries are associated with various complications during the perioperative period, with acute kidney injury (AKI) being a significant and not uncommon complication. AKI has been identified as an independent predictor of in-hospital mortality and can negatively impact patient outcomes, requiring close monitoring and prompt intervention^[^2^]^.

The incidence of AKI in patients undergoing cardiovascular surgery is reported to range between 10% and 40%^[^3^,^4^]^. The mechanisms leading to AKI in the context of cardiovascular surgery are multifactorial. Factors such as hemodynamic instability, the use of cardiopulmonary bypass (CPB), exposure to nephrotoxic agents, and pre-existing comorbidities contribute to the risk of AKI. Hemodynamic fluctuations during surgery can cause renal hypoperfusion, while inflammatory responses and oxidative stress can worsen renal injury. The use of contrast agents and certain medications after surgery also increases the risk of AKI^[^5^]^.

Given the significant impact of AKI on patient outcomes, early detection and targeted interventions are essential. The Kidney Disease: Improving Global Outcomes (KDIGO) criteria offer a standardized framework for classifying and managing AKI, updating and integrating definitions from the Risk, Injury, Failure, Loss, and End-stage kidney disease (RIFLE) and Acute Kidney Injury Network (AKIN) criteria. The KDIGO criteria are crucial for stratifying patients based on AKI severity and guiding clinical decisions effectively.

This study aims to evaluate the impact of postoperative AKI on mortality among patients who underwent coronary and/or heart valve surgery and required an extended stay in the intensive care unit (ICU).

## METHODS

### Study Design and Setting

This retrospective cohort study was conducted at a tertiary care center. The study period spanned from 2018 to 2022, including only patients who remained in the ICU for 72 hours or more after cardiovascular surgeries. A total of 507 patients were included in the final analysis: 261 patients underwent coronary artery bypass grafting, 206 had valve replacement surgeries, and the remaining 40 had combined CABG and valve replacement surgeries.

### Participants

The exclusion criteria were as follows: patients under 18 years old, pregnant women, nursing mothers, patients undergoing emergency surgeries, patients with incomplete medical records, those admitted to the ICU for reasons other than cardiovascular surgery, and individuals with a history of chronic renal failure. Additionally, patients who developed AKI outside the ICU were excluded to maintain a focused analysis of postoperative AKI in a controlled setting. After applying these criteria, 507 patients were included in the final analysis.

### Data Collection

Data were retrospectively collected from electronic medical records, including demographic information (age, sex) and postoperative outcomes (development of AKI, mortality). AKI was defined according to the KDIGO criteria, established in 2012. The KDIGO guidelines integrate and update previous definitions and classifications from the RIFLE and AKIN criteria to provide a unified standard. Urinary output data were not included in the analysis due to insufficient availability, which prevented accurate assessment. Patients were grouped based on the presence or absence of AKI and classified into stages 1, 2, and 3 according to the KDIGO criteria.

The primary outcome was in-hospital mortality. Secondary outcomes included time to extubation (hours), ICU days until ward transfer, and perioperative transfusion exposure (units of red blood cells [RBC], fresh frozen plasma [FFP], platelet/apheresis). Where recorded, initiation of renal replacement therapy (RRT) was assessed exploratorily. Outcomes were also summarized by KDIGO stages^[^1^-^3^]^.

The KDIGO guideline provides a well-structured framework for classifying and managing AKI.

Stage 1 AKI is characterized by a serum creatinine increase of ≥ 0.3 mg/dl (≥ 26.5 µmol/l) within 48 hours or an increase to ≥ 1.5 to two times the patient's baseline, which is known or presumed to have occurred within the last seven days. A urine output of < 0.5 ml/kg/h for six to 12 hours also falls under Stage 1.

Stage 2 AKI is defined by a further increase in serum creatinine to two to three times the baseline. In terms of urine output, < 0.5 ml/kg/h for more than 12 hours indicates this intermediate stage of AKI.

Stage 3, the most severe form of AKI, is indicated by a serum creatinine increase to three times the baseline, or an increase to ≥ 4.0 mg/dl (≥ 353.6 µmol/l), or the initiation of renal replacement therapy. For urine output, < 0.3 ml/kg/h for 24 hours or anuria for 12 hours also categorizes AKI as Stage 3^[^6^]^.

### Transfusion Practices

Perioperative transfusions (RBC, FFP, platelet/apheresis, whole blood) followed institutional protocols aligned with contemporary Society if Thoracic Surgeons/European Association for Cardio-Thoracic Surgery patient blood management guidance. Thresholds were restrictive and context-specific (*e.g.*, RBC at ~7-8 g/dL when stable, higher if ongoing ischemia/bleeding)^[^7^]^. Detailed orders were clinician-driven; therefore, we report exposures as units transfused rather than prescriptive algorithms.

### Data Processing

Patient demographics, clinical parameters, and outcomes were extracted from electronic medical records. Key variables included age, body mass index (BMI), sex, hypertension (HT), diabetes mellitus (DM), chronic obstructive pulmonary disease (COPD), preoperative ejection fraction (EF), erythrocyte suspension (ES), FFP, apheresis, and whole blood transfusions given in the ICU, extubation time, discharge to service days, preoperative lactate levels, aortic clamp time, and survival status.

Data processing involved several steps:

• Data Extraction: Patient data were extracted from electronic medical records.
• Data Cleaning: The extracted data were reviewed for completeness and accuracy. Missing data points were noted, and patients with incomplete records for key variables were excluded. Due to the retrospective nature of the study and the inability to accurately impute values, missing data were handled by exclusion.
• Data Categorization: Patients were categorized based on the presence or absence of postoperative AKI and further classified into stages 0, 1, 2, and 3 according to the KDIGO guidelines. The AKI criteria were based on the KDIGO standards, which integrate and update previous definitions and classifications from the RIFLE and AKIN criteria. For clarity, we use 'KDIGO 0' to denote patients without AKI.
• Handling of Outliers: Outliers were identified using standard statistical techniques and were either excluded or treated based on their impact on the overall data distribution.

### Statistical Analysis

We examined the association between AKI and in-hospital mortality using multivariable logistic regression, adjusting for age, sex, HT, DM, aortic cross-clamping (ACC) time, intraoperative transfusion and ICU transfusion exposures, and post-CPB inotropic support. Multicollinearity was assessed (variance inflation factor < 5). Results are reported as adjusted odds ratios (OR) with 95% confidence intervals. Two-sided *P* < 0.05 was considered statistically significant; very small values are reported as *P* < 0.001. When the omnibus one-way analysis of variance (ANOVA) was significant, post-hoc pairwise comparisons with appropriate correction were performed to identify differing groups.

Categorical variables were summarized as counts and percentages, while continuous variables were presented as means ± standard deviations or medians with interquartile ranges, depending on their distribution. The normality of continuous variables was assessed using the Shapiro-Wilk test.

Independent sample *t*-tests were used to compare means between the No-AKI and AKI groups for normally distributed variables. At the same time, the Mann-Whitney U test was applied for non-normally distributed variables. Fisher's exact test was used for categorical variables with small sample sizes to determine statistical significance, while chi-square tests were applied for other categorical comparisons. One-way ANOVA was used to compare means across KDIGO stages (0, 1, 2, 3).

All analyses were conducted using IBM SPSS Statistics for Windows, version 26 (IBM Corp., Armonk, N.Y., USA).

### Ethical Considerations

The Institutional Review Board (IRB) reviewed and approved the study protocol (IRB number 2023.06.74), which waived the requirement for informed consent due to the study's retrospective nature. All procedures followed the ethical standards of the committee responsible for human experimentation and the Helsinki Declaration. Measures were taken to ensure data privacy and confidentiality throughout the study.

## RESULTS

A total of 507 patients were included in the study, of whom 132 (26%) developed AKI postoperatively, while 375 (74%) did not. Significant differences were observed in several clinical parameters between the AKI and No-AKI groups. The AKI group exhibited a notably higher incidence of HT and DM compared to those without AKI (No-AKI: 152/375, AKI: 79/132, *P* < 0.001 and No-AKI: 227/375, AKI: 95/132, *P* = 0.021, respectively) (Table 1).

**Table 1:** Demographic and clinical characteristics of patients.

	No-AKI	AKI	*P*-value
	(n = 375)	(n = 132)	
Age	65.81 ± 12.30^a^	71.08 ± 10.78^a^	< 0.001^c^
BMI	27.81 ± 4.48^a^	28.19 ± 4.76^a^	0.550^c^
Sex (female/male)	135/240^b^	52/80^b^	0.529^d^
HT	152/375^b^	79/132^b^	< 0.001^d^
DM	227/375^b^	95/132^b^	0.021^d^
COPD	323/375^b^	120/132^b^	0.173^e^

a Mean ± standard deviation;

b Numbers;

c Independent sample *t*-test;

d Fisher's exact test;

e Chi-square test

BMI=body mass index; COPD=chronic obstructive pulmonary disease; DM=diabetes mellitus; HT=hypertension

The mean age of patients in the No-AKI group was significantly lower than that in the AKI group (65.81 ± 12.30 *vs.* 71.08 ± 10.78 years, *P* < 0.001). The mean BMI was similar in both groups (27.81 ± 4.48 *vs.* 28.19 ± 4.76, *P* = 0.550) (Table 1).

Sex distribution did not significantly differ between the groups (No-AKI: 135 females and 240 males; AKI: 52 females and 80 males; *P* = 0.529). The occurrence of COPD did not significantly differ between the groups (No-AKI: 323/375; AKI: 120/132; *P* = 0.173) (Table 1).

There was no significant difference in the preoperative EF between the No-AKI and AKI groups (51.88 ± 10.99 *vs.* 51.86 ± 10.24, *P* = 0.987).

Mean RBC unit transfusion was significantly higher in the AKI group compared to the No-AKI group (4.03 ± 4.98 *vs.* 1.72 ± 1.94 units, *P* < 0.001). The mean volume of FFP transfused and the requirement for apheresis were significantly higher in the AKI compared to the No-AKI group (2.23 ± 2.75 *vs.* 1.11 ± 1.60 and 0.59 ± 1.25 *vs.* 0.11 ± 0.47, respectively; *P* < 0.001) (Table 2). Additionally, the number of whole blood units used was significantly higher in the AKI group compared to the No-AKI group (0.15 ± 0.47 *vs.* 0.04 ± 0.32 units, respectively, *P* = 0.016) (Table 2).

**Table 2:** Comparison of potential causes and consequences of acute kidney injury (AKI).

	No-AKI	AKI	*P*-value
	(n = 375)	(n = 132)	
Preoperative ejection fraction	51.88 ± 10.99^a^	51.86 ± 10.24^a^	0.987^c^
Erythrocyte suspension (unit)	1.72 ± 1.94^a^	4.03 ± 4.98^a^	< 0.001^c^
Fresh frozen plasma (unit)	1.11 ± 1.60^a^	2.23 ± 2.75^a^	< 0.001^c^
Apheresis (unit)	0.11 ± 0.47^a^	0.59 ± 1.25^a^	< 0.001^c^
Whole blood (unit)	0.04 ± 0.32^a^	0.15 ± 0.47^a^	0.016^c^
Extubation (hours)	20.60 ± 19.73^a^	63.95 ± 73.92^a^	< 0.001^c^
Discharge to service (days)	3.49 ± 5.85^a^	6.92 ± 9.97^a^	0.010^c^
Preoperative lactate level	1 ± 0.39^a^	1.10 ± 0.47^a^	0.499^c^
Aortic cross-clamping time	129.79 ± 187.86^a^	145.54 ± 167.39^a^	0.522^c^
Mortality	38/375^b^	29/132^b^	< 0.001^d^
ACEF scoree	1.34 ± 0.41	1.48 ± 0.43	0.004

a Mean ± standard deviation;

b Numbers;

c Independent sample t-test;

d Fisher's exact test

ACEF=Age, Creatinine, Ejection Fraction

Patients with AKI had significantly prolonged extubation times (63.95 ± 73.92 hours *vs.* 20.60 ± 19.73 hours, *P* < 0.001) and longer hospital stays until discharge to the service (6.92 ± 9.97 days *vs.* 3.49 ± 5.85 days, *P* = 0.010) compared to those without AKI (Table 2).

There was no significant difference in preoperative lactate levels between the groups (1.00 ± 0.39 in No-AKI *vs.* 1.10 ± 0.47 in AKI, *P* = 0.499). Similarly, the ACC time did not differ significantly between the No-AKI and AKI groups (129.79 ± 187.86 *vs.* 145.54 ± 167.39, *P* = 0.522) (Table 2).

Lastly, the survival rate was significantly higher in the No-AKI group compared to the AKI group (347/375 *vs.* 103/132, *P* < 0.001) (Table 2).

The ANOVA revealed no significant difference in preoperative EF among the KDIGO stages (F = 0.75, *P* = 0.521). Extubation time, however, showed a significant increase with higher KDIGO stages, indicating longer mechanical ventilation duration in patients with more severe AKI (F = 27.96, *P* < 0.001). Similarly, the length of stay before discharge to the service was significantly prolonged in patients with higher KDIGO stages (F = 3.801, *P* = 0.023) (Table 3).

**Table 3:** Results for key clinical variables based on Kidney Disease: Improving Global Outcomes (KDIGO) stages.

	KDIGO Stage	N	Mean ± SD	F-value	*P*-value	Significant Differences Between Groups
Preoperative EF (%)	No AKI patients	375	51.88 ± 10.99	0.75	0.521	None
	1	83	51.19 ± 8.74			
	2	36	51.42 ± 9.67			
	3	13	56.25 ± 11.11			
Extubation time (hours)	No AKI patients	375	20.60 ± 19.73	27.96	< 0.001	KDIGO 0 *vs.* 1, 2, 3
	1	96	59.72 ± 10.54			
	2	23	75.00 ± 12.47			
	3	13	72.86 ± 9.32			
Discharge to service (days)				3.801	0.023	
Erythrocyte suspension	No AKI patients	375	1.72 ± 1.94	17	< 0.001	KDIGO 0, 3 *vs.* 1, 2
	1	96	4.61 ± 1.02			
	2	23	3.00 ± 0.89			
	3	13	1.71 ± 0.77			
Fresh frozen plasma	No AKI patients	375	1.11 ± 1.60	11.71	< 0.001	KDIGO 0, 2, 3 *vs.* 1
	1	96	2.73 ± 0.67			
	2	23	1.15 ± 0.39			
	3	13	0.57 ± 0.23			
Apheresis (units)	No AKI patients	375	0.11 ± 0.47	12.90	< 0.001	KDIGO 0, 3 *vs.* 1, 2
	1	96	0.75 ± 0.21			
	2	23	0.23 ± 0.10			
	3	13	0.14 ± 0.05			
Whole blood (units)	No AKI patients	375	0.04 ± 0.32	2.62	0.051	None
	1	96	0.16 ± 0.05			
	2	23	0.23 ± 0.07			
	3	13	0.00 ± 0.00			
Aortic cross-clamping time (minutes)	No AKI patients	375	129.79 ± 187.86	2.31	0.076	None
	1	96	133.11 ± 30.22			
	2	23	134.08 ± 45.67			
	3	13	140.33 ± 15.21			
Preoperative lactate				0.459	0.499	

a One-way analysis of variance was used

AKI=acute kidney injury; EF=ejection fraction; SD=standard deviation

Apheresis requirements increased with higher KDIGO stages (F = 12.90, *P* < 0.001) (Table 3).

Among patients with AKI, the distribution across KDIGO stages 1 - 3 is presented in Table 3.

In the multivariable logistic regression analysis including age, sex, HT, DM, ACC time, ICU transfusion, intraoperative transfusion, and post-CPB inotrope support, only AKI was significantly associated with in-hospital mortality. The presence of AKI increased the risk of death by approximately 2.7-fold (B = 1.009, *P* = 0.017, OR = 2.742) (Table 4).

**Table 4:** Logistic regression analysis of risk factors for in-hospital mortality.

	B	S.E.	Wald chi-square statistic	df	*P*-value^*^	OR (Exp(B))
Age	0.032	0.019	2.661	1	0.103	1.032
Sex (0 = female, 1 = male)	0.583	0.412	2.009	1	0.156	1.792
Hypertension	0.593	0.482	1.509	1	0.219	1.809
Diabetes mellitus	-0.313	0.444	0.497	1	0.481	0.731
Aortic cross-clamping time	0.000	0.001	0.185	1	0.667	1.000
AKI	1.009	0.423	5.684	1	0.017	2.742
ICU transfusion	-0.390	0.460	0.722	1	0.396	0.677
Intraoperative transfusion	0.573	0.487	1.379	1	0.240	1.773
Post-CPB inotrope support	-0.687	0.511	1.811	1	0.178	0.503
Constant	-4.720	1.467	10.345	1	0.001	0.009

* Logistic regression test has been used

AKI=acute kidney injury; B=regression coefficient; CPB=cardiopulmonary bypass; df=degrees of freedom; ICU=intensive care unit; OR=odds ratio; S.E.=standart error

The observed association between AKI and greater transfusion exposure warrants cautious interpretation. Confounding by indication (*e.g.*, bleeding leading to transfusion which in turn predisposes to AKI) and potential reverse causality (*e.g.*, AKI-related coagulopathy or hemodilution increasing transfusion needs) may operate concurrently. Although our multivariable models were adjusted for operative times and transfusion variables, the observational design precludes causal inference, and residual confounding is likely.

### Limitations

Urine-output data were incompletely available; therefore, AKI was defined and staged using creatinine-based KDIGO criteria. We acknowledge this as a limitation since oliguria forms part of KDIGO definitions and may improve sensitivity for early AKI. We lacked granular data on nephrotoxic drug exposure (*e.g.*, contrast media, nonsteroidal anti-inflammatory drugs [NSAIDs], aminoglycosides) and vasoactive dose/duration; this may introduce residual confounding. Detailed baseline renal function (estimated glomerular filtration rate categories) and EF (or Age, Creatinine, Ejection Fraction [ACEF] score) could not be included in the final multivariable model due to missingness; therefore, residual confounding cannot be excluded.

## DISCUSSION

The main findings of this study show that postoperative AKI significantly affects patient outcomes after cardiovascular surgery, especially in those needing extended stays in the ICU. We found that AKI is linked to higher death rates, longer extubation times, and longer hospital stays. Also, the severity of AKI, graded by the KDIGO stages, is connected to a greater need for blood transfusions and a higher occurrence of HT and DM among affected patients. These findings highlight the urgent need for early detection and treatment of AKI to improve survival and recovery in this high-risk group.

Jiang et al.^[^8^]^ found no significant difference in sex distribution between the AKI and non-AKI groups in the normal population, which is consistent with our findings. The prevalence of HT and DM was significantly higher in the AKI group. This aligns with previous studies identifying DM as an independent risk factor for AKI onset^[^8^]^. Additionally, HT, a common comorbidity in patients with AKI, contributes to an increased risk of kidney injury in both the general population and cardiovascular surgery patients^[^9^,^10^]^.

Regarding COPD prevalence, our study did not reveal a significant difference between the AKI and non-AKI groups. COPD has been identified as a risk factor for AKI. According to the study's findings, the incidence of AKI in COPD patients was 128 per 100,000 person-years, which is higher than in the general population^[^11^]^. Another study did not find a significant connection, highlighting the complexity and inconsistency in the relationship between these two conditions (7.5% [No-AKI] *vs.* 5.7% [AKI], *P* = 1)^[^12^]^.

Preoperative EF did not significantly differ between patients with and without AKI, indicating that baseline cardiac function was similar across groups. This finding aligns with some studies that found no significant effect of EF on AKI development. However, other research suggests a possible but not definitively established link between lower EF and increased AKI risk^[^13^-^15^]^.

Additionally, the ACEF score, which uses EF as a key component, is a useful predictor of AKI in high-risk patients undergoing cardiovascular procedures^[^16^]^. Avci et al.^[^13^]^ showed that a higher ACEF score was linked to an increased risk of AKI after endovascular aortic repair, highlighting the importance of considering age and baseline kidney function along with EF when evaluating AKI risk. Our results agree with this, indicating that while EF alone might not be a strong independent predictor, including it in combined scores like ACEF improves its prognostic value for AKI risk assessment.

The ACEF score was updated to ACEF II by adding factors like emergency surgery and preoperative anemia (hematocrit < 36%) into the risk assessment. This new version showed better predictive performance than the original ACEF I score^[^17^]^.

Ibrahim et al.^[^18^]^ reported that patients with AKI required significantly more ES, FFP, and apheresis in their study on surgical valve replacement, which is consistent with our findings.

In our cohort, ES, FFP, and platelet suspension were administered to patients based on clinical need, following established guidelines. We observed that patients with worsening AKI tended to receive higher volumes of these blood components. This finding is consistent with similar trends reported in the literature.

The development of AKI is associated with prolonged postoperative complications, increased in-hospital stay, and significant rises in healthcare costs^[^19^,^20^]^. Regarding extubation time, our study found that patients with AKI experienced prolonged mechanical ventilation, which is consistent with the findings of Ibrahim et al.^[^18^]^. This suggests that AKI may exacerbate the postoperative course, leading to delayed weaning from mechanical ventilation. The extended ICU stay before discharge to the service, reported by Ibrahim et al.^[^18^]^, further supports the notion that AKI complicates recovery, prolonging hospitalization and increasing the burden on healthcare resources.

Ibrahim et al.^[^18^]^ categorized patients based on ACC time (< 90 min and > 90 min) and pump time (< 120 min and > 120 min) in their study, demonstrating that both ACC and pump time were significant predictors of AKI. In our cohort, the mean ACC time was longer compared to the reference by Ibrahim et al.^[^18^]^, which could be attributed to the complexity of the procedures performed in our study, such as more extensive surgeries or additional interventions. We must highlight that some of our patients underwent a combination of methods, including valve replacements and complex CABG, which may explain the extended clamp time.

Ibrahim et al.^[^18^]^ noted no significant differences between patients with and without AKI regarding preoperative lactate levels, similar to our findings.

Studies have shown a strong link between postoperative AKI and higher mortality rates in patients undergoing cardiovascular surgery, which aligns with our findings^[^21^,^22^]^. This association is consistent with existing literature, identifying AKI as a key predictor of adverse postoperative outcomes, including longer mechanical ventilation, extended ICU stays, and increased hospital mortality^[^23^]^. Our study further emphasizes that preventing or reducing AKI is crucial for improving surgical outcomes, especially in high-risk cardiovascular patients.

Although our study did not specifically address management strategies for AKI, it is essential to integrate predictive, preventative, and therapeutic approaches into clinical practice. As emphasized by Cheruku et al.^[^10^]^, effective management of AKI after cardiac surgery involves early prediction using biomarkers, preventive measures such as optimizing fluid management, and timely intervention to reduce the risks associated with AKI.

Numerous studies have examined the impact of AKI on mortality after cardiovascular surgery. Research shows that AKI may raise postoperative mortality through various pathophysiological processes, including inflammation, oxidative stress, and endothelial dysfunction. However, the exact extent of this effect and its underlying mechanisms are still not fully understood^[^24^]^.

The connection between AKI after surgery and its effect on mortality is a major clinical concern. Our research and previous studies emphasize the importance of renal function in determining patient outcomes after cardiac surgery. AKI has consistently been shown as a key factor linked to higher mortality in these patients^[^24^,^25^]^.

Yaqub et al.^[^26^]^ compared KDIGO, AKIN, and RIFLE criteria for diagnosing AKI and predicting outcomes after cardiac surgery. The study concluded that AKI was a common occurrence following elective cardiac surgeries (CABG and heart valve surgeries).

The management of AKI in patients who undergo cardiovascular surgery, especially those with prolonged ICU stays, is guided by the latest KDIGO guidelines, which focus on both preventive and treatment strategies. Preoperative optimization of renal function is crucial, particularly for high-risk patients such as those with existing chronic kidney disease, diabetes, or HT^[^6^]^. Perioperatively, efforts aim to maintain hemodynamic stability and reduce renal hypoperfusion through optimal fluid management and careful use of vasopressors. Intraoperative factors like minimizing CPB time and closely monitoring renal perfusion are vital to lowering the risk of AKI^[^12^]^. Additionally, avoiding nephrotoxic agents (*e.g.*, contrast media, NSAIDS) is essential, and early detection of AKI through biomarkers such as serum creatinine is emphasized^[^12^]^.

Postoperatively, management involves monitoring fluid status, electrolyte imbalances, and acid-base disturbances. Diuretics are used cautiously to control fluid overload. Simultaneously, RRT is initiated for patients with refractory volume overload, severe electrolyte imbalances (*e.g.*, hyperkalemia), or acidosis that do not respond to medical treatment. Blood transfusion practices follow established cardiac surgery protocols, and transfusions are given only when necessary to prevent overloading the already stressed kidneys. The KDIGO guidelines also recommend early intervention based on AKI severity, enabling stratified care and better outcomes. Early detection and treatment of AKI in cardiovascular surgery patients are crucial for reducing mortality and enhancing postoperative recovery^[^6^]^.

### Limitations

Our study's limitations include its retrospective design and single-center setting, which may limit the generalizability of the findings. In our research, biomarkers such as creatinine, blood urea nitrogen, and glomerular filtration rate were evaluated, but results for other biomarkers like neutrophil gelatinase-associated lipocalin, kidney injury molecule-1, liver-type fatty acid-binding protein, interleukin-18, insulin-like growth factor-binding protein 7, tissue inhibitor of metalloproteinase 2, and calprotectin were not available. Significant indicators of kidney function, such as urine output, were not assessed and, therefore, could not be included^[^27^]^.

## CONCLUSION

AKI remains a significant complication after cardiovascular surgeries, greatly affecting patient outcomes, especially in those needing prolonged ICU stays. Our findings show that AKI not only extends ICU length of stay but also raises in-hospital mortality, underscoring the importance of early detection and improved perioperative renal protection strategies. The multifactorial causes of AKI in this patient group require a comprehensive approach, combining hemodynamic management, careful fluid control, and avoidance of nephrotoxic agents.

Furthermore, using standardized AKI classification systems like KDIGO criteria helps with more accurate risk assessment and targeted treatments. Future research should aim to find predictive biomarkers and improve perioperative protocols to lower AKI risk and enhance long-term kidney health. As cardiovascular surgical patients become more complex, an interdisciplinary approach to postoperative care is essential for reducing AKI-related complications and death.

These findings underscore the importance of continuous monitoring and individualized patient management in intensive care settings, reinforcing the necessity of evidence-based strategies for improving both shortand long-term outcomes in critically ill cardiac surgery patients.

## Data Availability

The authors declare that the data are not publicly available due to patient privacy and confidentiality restrictions. Data may be available from the corresponding author upon reasonable request.

## Artificial Intelligence Usage

The authors declare use of ChatGPT (OpenAI) to assist with English language editing and translation of the manuscript. The content produced by the artificial intelligence tool was revised and edited by the authors as necessary, and they take full responsibility for the content to be published.
